# Supplementary material for: The genome sequence of the Catalonian Wall Lizard, Podarcis liolepis (Boulenger, 1905) (Squamata: Lacertidae)
Source: Wellcome Open Res. 2025 Sep 10;10:505. [Version 1] doi: 10.12688/wellcomeopenres.24854.1 (PMC12572770; doi:10.12688/wellcomeopenres.24854.1)
Supplement: Peer review history

# Reviewer report: Reviewer response for version 1

**DOI:** 10.21956/wellcomeopenres.27378.r133063

**Published:** 2025-10-29

**Authors:** Kathryn R. Elmer

**Affiliations:** 1 University of Glasgow, Glasgow, UK

This communication describes the sequencing and assembly of a new genome for the wall lizard. The approach follows the well established Tree of Life pipeline for both primary material and analysis of the sequence data. One haplotype is generated that includes the anticipated number of chromosomes including the Z and W and the other Haplotype to comparable scaffold quality. Made available, the data will be useful for a range of evolutionary and comparative biology applications. The details are sufficient for recreating the methods.

 minor:

 There seems to be a duplication of text related to the shearing method and DNA extraction

 Is it correct that the HiC done on ethanol preserved tissue that was then frozen?

Are sufficient details of methods and materials provided to allow replication by others?

Yes

Is the rationale for creating the dataset(s) clearly described?

Yes

Are the datasets clearly presented in a useable and accessible format?

Yes

Are the protocols appropriate and is the work technically sound?

Yes

Reviewer Expertise:

evolutionary biology

I confirm that I have read this submission and believe that I have an appropriate level of expertise to confirm that it is of an acceptable scientific standard.

# Reviewer report: Reviewer response for version 1

**DOI:** 10.21956/wellcomeopenres.27378.r134271

**Published:** 2025-09-29

**Authors:** Hong-Xin Xie

**Affiliations:** 1 University of Glasgow, Glasgow, UK

The data note provides a de novo haplotype-resolved assembly of the Catalonian Wall Lizard, *Podarcis liolepis.* The presented methods are appropriate and of sufficient detail.

 The presented assembly is highly continuous and of high quality as assessed by multiple methods, and especially, fully meets the EBP proposed reference genome standard.

 The presented tables and figure plots are also clear and well organized. This is a piece of excellent-written work that I enjoyed.

Are sufficient details of methods and materials provided to allow replication by others?

Yes

Is the rationale for creating the dataset(s) clearly described?

Yes

Are the datasets clearly presented in a useable and accessible format?

Yes

Are the protocols appropriate and is the work technically sound?

Yes

Reviewer Expertise:

evolutionary genomics, especially focusing on squamate reptiles.

I confirm that I have read this submission and believe that I have an appropriate level of expertise to confirm that it is of an acceptable scientific standard.
